# Evidence-based uncertainty: why equipoise is essential in clinical research

**DOI:** 10.36416/1806-3756/e20260090

**Published:** 2026-03-05

**Authors:** Flávia Fonseca Fernandes, Cecilia Maria Patino, Juliana Carvalho Ferreira

**Affiliations:** 1. Methods in Epidemiologic, Clinical, and Operations Research-MECOR-program, American Thoracic Society/Asociación Latinoamericana del Tórax, Montevideo, Uruguay.; 2. Departamento de Medicina, Universidade Federal de Catalão, Catalão (GO), Brasil.; 3. Department of Preventive Medicine, Keck School of Medicine, University of Southern California, Los Angeles (CA) USA.; 4. Divisão de Pneumologia, Instituto do Coração, Hospital das Clínicas Faculdade de Medicina, Universidade de São Paulo, São Paulo (SP), Brasil.

## ILLUSTRATIVE SCENARIO

A 55-year-old woman with non-cystic fibrosis bronchiectasis has daily sputum production and recurrent exacerbations despite optimized airway clearance and guideline-based preventive measures. Frustrated by persistent symptoms and repeated antibiotic courses, she asks her doctor about a novel inhaled antibiofilm agent that is currently being tested in a randomized clinical trial. The patient assumes that joining the clinical trial implies receiving the investigational drug and improving her clinical condition. The doctor pauses to reflect before formulating an answer: the very reason the clinical trial is being performed is that clinicians and researchers continue to face uncertainty about the tested intervention and control treatments-and current evidence does not establish which approach is superior.

## WHAT IS EQUIPOISE?

Equipoise is genuine, rational clinical uncertainty regarding whether one of two (or more) interventions is superior for a given clinical condition. It reflects a reasoned assessment that the available evidence does not allow the medical community to conclude that one option is clearly better than the other.^1^

## WHY DO RANDOMIZED TRIALS DEPEND ON IT?

Randomization is ethically defensible only under conditions of equipoise. If superiority were already established-or could be reasonably defended as established-allocating patients by chance to receive the intervention or a control treatment (placebo or standard of care) would be ethically problematic. Thus, randomized clinical trials presuppose equipoise, serving as a method to resolve clinically meaningful uncertainty and justify randomization.^1^

## INDIVIDUAL VS. COMMUNITY EQUIPOISE

Clinicians rarely remain entirely neutral regarding the efficacy of new treatments or interventions. Even when acknowledging uncertainty, they may hold preferences informed by pathophysiological reasoning, preliminary data, or prior clinical experience. This corresponds to individual equipoise (or its absence): the uncertainty or preference of a single clinician.

To address the practical limitations of relying on individual neutrality, the classic proposal shifts attention to the collective: clinical (community) equipoise, defined as honest and reasonable disagreement among informed experts regarding which intervention is superior for a given clinical condition. In practical terms, a clinical trial may remain justified even when some clinicians hold preferences, provided that the expert community has not reached a defensible consensus that one intervention is superior to the other(s).^2^

**Figure 1:**
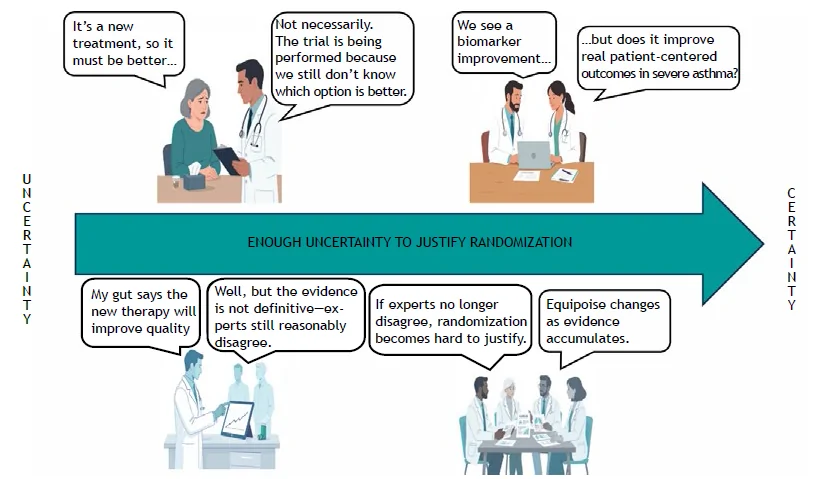
Clinical equipoise in different perspectives. Source: Adapted from Alexander JH.^3^.

In summary, while individual equipoise asks, “Am I personally uncertain about the superiority of this intervention?”, community equipoise asks, “Is the expert community still reasonably uncertain?”-and it is this latter standard that supports the ethical and practical feasibility of randomized clinical trials (Figure 1).

## THE DYNAMIC NATURE OF UNCERTAINTY

Recognizing equipoise helps distinguish clinical optimism from empirical certainty and clarifies the role of randomized clinical trials as instruments for resolving uncertainty at the level of the expert community. In this framework, clinical trials are not performed to confirm what is already known, but to generate sufficiently robust evidence to settle questions that remain open and clinically relevant. Importantly, equipoise is not static and should be reassessed as new information emerges (Figure 1).

In the case of the patient described in the practical scenario, acknowledging community equipoise provides the clinician with a clear rationale to explain that the trial is being performed precisely because the expert community remains uncertain about the efficacy of the new drug. This ensures that the decision to participate is grounded in scientific reality rather than the assumption that “new treatment” is synonymous with “better treatment”.

### KEY POINTS

- Equipoise is rational, community-level uncertainty about which intervention is better for a given clinical condition.
- Randomized clinical trials depend on equipoise: if superiority is already reasonably established, randomization is hard to justify.
- In practice, equipoise is most often challenged by overinterpretation of early data and by confusing “new” with “better.”
- Equipoise is not static and should be readdressed as new information emerges.
